# Complete genome sequences of eight *Caryophanales* strains isolated from the rhizosphere of strawberry in Canada

**DOI:** 10.1128/mra.00482-25

**Published:** 2025-07-31

**Authors:** Adrien Biessy, Martin Filion

**Affiliations:** 1 Department of Plant Science, McGill University, MacDonald Campus 151165, Sainte-Anne-de-Bellevue, Québec, Canada; The University of Arizona 8041, Tucson, Arizona, USA

**Keywords:** *Caryophanales*, rhizosphere

## Abstract

Here, we present the complete genome sequences of eight bacterial strains isolated from the rhizosphere of strawberry plants grown in an agricultural field located in Bouctouche, New Brunswick, Canada. The strains belong to four different genera (*Bacillus*, *Lysinibacillus*, *Psychrobacillus*, and *Priestia*) within the order *Caryophanales*.

## ANNOUNCEMENT

The genus *Bacillus* (order *Caryophanales*) is an important genus of endospore-forming rod-shaped bacteria that includes both human pathogens and plant-beneficial strains (1, 2). Over the years, several genera were created from species previously included in this genus, such as *Lysinibacillus*, *Psychrobacillus*, and *Priestia* (3–5). Many of these genera also include strains with plant growth promotion activity (6, 7). Genome sequencing of plant-beneficial strains belonging to the genus *Bacillus* and its related genera will promote the development of microbial biofertilizers and contribute to a better understanding of the plant growth promotion molecular mechanisms involved. Here, we report the complete genome sequences of eight *Caryophanales* strains.

The strains were isolated in 2004 from the rhizosphere of strawberry plants (*Fragaria* x *ananassa* Duch.) grown in an agricultural field located in Bouctouche, New Brunswick, Canada (46.432232,-64.769877) using a protocol described previously (8). Briefly, rhizosphere soil samples were collected and kept at 4°C. One gram of soil was suspended in 100 mL of a 0.9% NaCl solution and agitated for 5 min at 250 rpm. The suspension was serially diluted and plated onto tryptic soy agar plates (BD Difco), which were incubated at 25°C for 48 h. Isolated colonies were subsequently purified on the same medium. Bacterial strains were cryopreserved at −80°C in sterile distilled water supplemented with 25% (v/v) glycerol.

Bacterial strains were grown directly from frozen stocks in tryptic soy broth (BD Difco) for 24 h at 25°C, and genomic DNA was extracted using the DNeasy UltraClean Microbial kit (Qiagen) following the manufacturer’s instructions. Library preparation and genome sequencing were performed at the Integrated Microbiome Resource (Halifax, NS, Canada). Genomic DNA was mechanically sheared and size-selected using g-TUBE devices (Covaris). The resulting DNA fragments (~8-10 kb) were converted into a WGS library using the SMRTbell Prep Kit 3.0 (Pacific Biosciences) as per the manufacturer’s instructions. Genome sequencing was performed on a Sequel II sequencer (Pacific Biosciences) loaded with a SMRT Cell 8M (v2.0 chemistry). FastQC v0.11.9 (9) was used to check the quality of the raw reads. Genome assembly was performed with Flye v2.9-b1768 using the command “--pacbio-hifi” (HiFi reads) or “--pacbio-raw” (CLR reads). Chromosome and plasmid sequences were automatically circularized by the Flye algorithm and rotated using Geneious Prime v2025.1.2 (Biomatters). The origin of the chromosome sequences was set at the start of the *dnaA* gene. The genomes were annotated by the NCBI Prokaryotic Genome Annotation Pipeline v6.10 (10). Default parameters were used for all software, unless otherwise specified. Sequencing, assembly metrics, and genome features are presented in Table 1.

**TABLE 1:** Genome sequencing/assembly metrics and genome features

Metrics	Strains							
	LBUM116	LBUM136	LBUM169	LBUM342	LBUM349	LBUM447	LBUM564	LBUM632
No. of CLR reads	3,921,600	2,573,417	25,834,315	1,750,168	15,855,285	4,072,462	1,794,769	2,497,778
CLR read N50 (bp)	4,877	5,896	4,200	5,046	2,408	4,848	5,932	6,045
No. of HiFi reads	96,083	70,512	544,216	42,597	232,162	94,911	49,220	72,518
HiFi read N50 (bp)	6,693	7,977	6,829	7,193	4,065	6,804	8,364	8,196
Reads used for assembly	HiFi	HiFi	HiFi	CLR	CLR	HiFi	HiFi	CLR
Coverage (×)	103	70	461	1,107	4,517	80	76	1,708
Genome size (bp)	4,731,136	5,911,531	5,544,785	5,416,514	6,123,168	5,968,817	4,009,808	6,065,531
G + C content (%)	37.6	35.3	35.6	36.8	37.6	35.3	36.2	37.6
No. of chromosomes	1	1	1	1	1	1	1	1
No. of plasmids	0	2	7	1	8	5	0	10
No. of coding DNA sequences	4,539	5,952	5,528	5,158	6,190	6,035	3,884	6,085
No. of pseudogenes	59	239	405	118	95	288	45	102
No. of rRNAs	34	42	39	43	47	42	30	47
No. of tRNAs	107	107	107	110	144	107	74	152
Closest type strain (accession no.)	*Lysinibacillus fusiformis* ATCC 7055^T^ (NZ_PYWL00000000)	*Bacillus mycoides* DSM 2048^T^ (NZ_CM000742)	*Bacillus pseudomycoides* DSM 12442^T^ (NZ_CM000745)	*Lysinibacillus* *xylanilyticus* DSM 23493^T^ (NZ_LFXJ00000000)	*Priestia megaterium* ATCC 14581^T^ (NZ_CP069288)	*Bacillus mycoides* DSM 2048^T^ (NZ_CM000742)	*Psychrobacillus* *psychrodurans* DSM 11713^T^ (NZ_JAMKBK000000000NZ_JAMKBK000000000)	*Priestia megaterium* ATCC 14581^T^ (NZ_CP069288)
Digital DNA-DNA hybridization value (%)	97.1	70.8	68.5	69.3	72.7	93	79.6	71.2
Average nucleotide identity (%)	99.4	95.9	95.6	96	95.6	98.7	97.5	95.5
Identification	*Lysinibacillus fusiformis*	*Bacillus mycoides*	*Bacillus pseudomycoides*	*Lysinibacillus xylanilyticus*	*Priestia megaterium*	*Bacillus mycoides*	*Psychrobacillus* *psychrodurans*	*Priestia megaterium*
GenBank accession no.	CP189820	CP189817 CP189818 CP189819	CP189809 CP189810 CP189811 CP189812 CP189813 CP189814 CP189815 CP189816	CP189807 CP189808	CP189798 CP189799 CP189800 CP189801 CP189802 CP189803 CP189804 CP189805 CP189806	CP189792 CP189793 CP189794 CP189795 CP189796 CP189797	CP189791	CP189780 CP189781 CP189782 CP189783 CP189784 CP189785 CP189786 CP189787 CP189788 CP189789 CP189790
SRA accession no.	SRR33306138	SRR33306137	SRR33306136	SRR33306135	SRR33306134	SRR33306133	SRR33306132	SRR33306131

**Fig 1:**
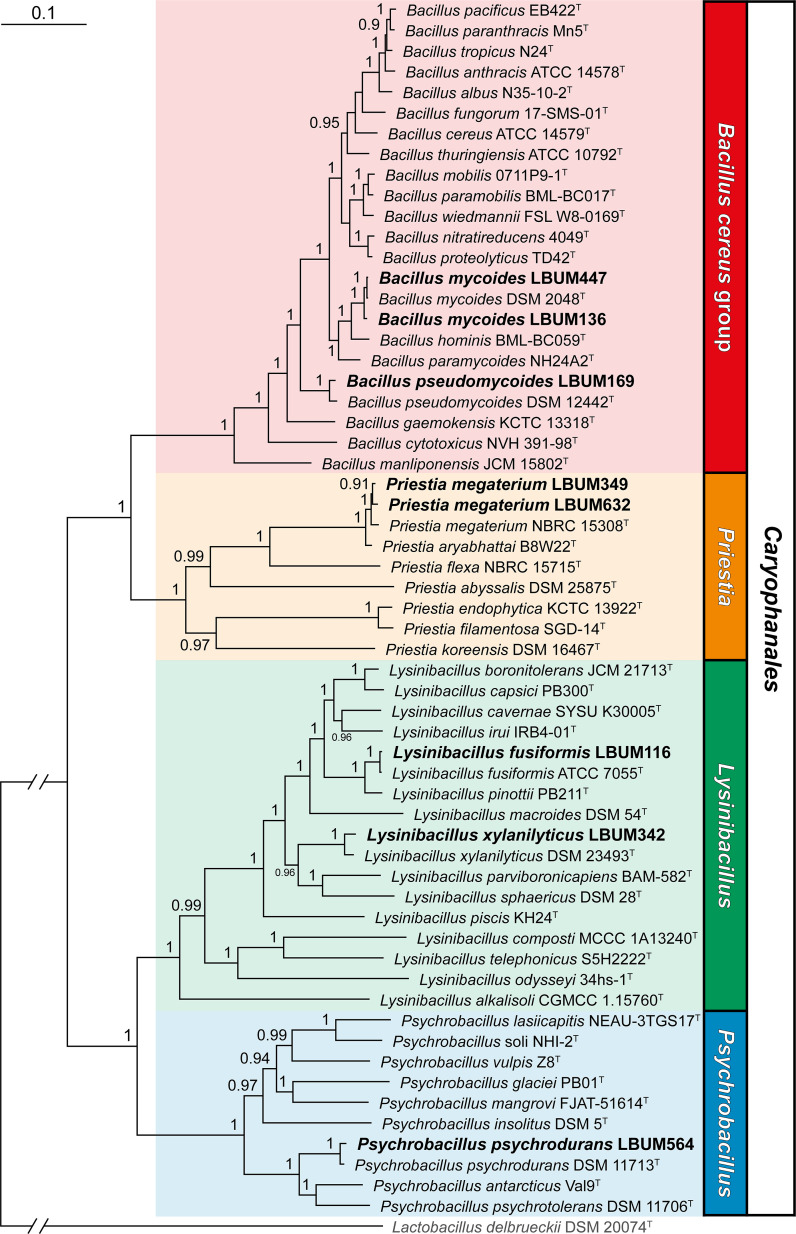
Phylogenetic relationships between eight *Caryophanales* strains and closely related type strains. The genomes of type strains belonging to the *B. cereus* group and the *Priestia*/*Psychrobacillus*/*Lysinibacillus* genera were downloaded from GenBank. The complete nucleotide sequences of four housekeeping genes (*gyrA*, *gyrB*, *rpoB*, and *rpoC*) were extracted from each genome, concatenated, and subsequently aligned using Clustal Omega v1.2.2 (13). The resulting alignment was used to generate a phylogenetic tree using FastTree v2.1 (14) and the generalized time reversible model. The tree topology was verified by computing Shimodaira Hasegawa support values (15). Only support values above 0.5 are displayed at the nodes. *Lactobacillus delbrueckii* DSM 20074^T^ was used as an outgroup. The eight strains under study are highlighted in bold. The scale bar indicates sequence divergence.

Genome size ranges from 4.01 to 6.12 Mb, and the G + C content fluctuates between 35.3% and 37.6%. The Type (Strain) Genome Server (11) and the JSpeciesWS web server (12) were used to identify the strains to the species level (Table 1). The strains belong to four different genera (*Bacillus*, *Lysinibacillus*, *Psychrobacillus*, and *Priestia*) within the order *Caryophanales*. To visualize the phylogenetic relationships between the eight strains and closely related type strains, we performed a multilocus sequence analysis (Fig. 1).

## Data Availability

The complete genome sequences of the eight bacterial strains (BioProject PRJNA1255136) have been deposited at GenBank. The versions described in this paper are the first versions. The raw sequencing data have been deposited to the Sequence Read Archive (SRA). Accession numbers are provided in Table 1.

## References

[1] Didelot X, Barker M, Falush D, Priest FG. 2009. Evolution of pathogenicity in the Bacillus cereus group. Syst Appl Microbiol 32:81–90. doi:10.1016/j.syapm.2009.01.001 19200684

[2] Miljaković D, Marinković J, Balešević-Tubić S. 2020. The significance of Bacillus spp. in disease suppression and growth promotion of field and vegetable crops. Microorganisms 8:1037. doi:10.3390/microorganisms8071037 32668676 PMC7409232

[3] Ahmed I, Yokota A, Yamazoe A, Fujiwara T. 2007. Proposal of Lysinibacillus boronitolerans gen. nov. sp. nov., and transfer of Bacillus fusiformis to Lysinibacillus fusiformis comb. nov. and Bacillus sphaericus to Lysinibacillus sphaericus comb. nov. Int J Syst Evol Microbiol 57:1117–1125. doi:10.1099/ijs.0.63867-0 17473269

[4] Krishnamurthi S, Ruckmani A, Pukall R, Chakrabarti T. 2010. Psychrobacillus gen. nov. and proposal for reclassification of Bacillus insolitus Larkin & Stokes, 1967, B. psychrotolerans Abd-El Rahman et al., 2002 and B. psychrodurans Abd-El Rahman et al., 2002 as Psychrobacillus insolitus comb. nov., Psychrobacillus psychrotolerans comb. nov. and Psychrobacillus psychrodurans comb. nov. Syst Appl Microbiol 33:367–373. doi:10.1016/j.syapm.2010.06.003 20650590

[5] Gupta RS, Patel S, Saini N, Chen S. 2020. Robust demarcation of 17 distinct Bacillus species clades, proposed as novel Bacillaceae genera, by phylogenomics and comparative genomic analyses: description of Robertmurraya kyonggiensis sp. nov. and proposal for an emended genus Bacillus limiting it only to the members of the Subtilis and Cereus clades of species. Int J Syst Evol Microbiol 70:5753–5798. doi:10.1099/ijsem.0.004475 33112222

[6] Biedendieck R, Knuuti T, Moore SJ, Jahn D. 2021. The “beauty in the beast”-the multiple uses of Priestia megaterium in biotechnology. Appl Microbiol Biotechnol 105:5719–5737. doi:10.1007/s00253-021-11424-6 34263356 PMC8390425

[7] Hilário S, Gonçalves MFM, Matos I, Rangel LF, Sousa JA, Santos MJ, Ayra-Pardo C. 2024. Comparative genomics reveals insights into the potential of Lysinibacillus irui as a plant growth promoter. Appl Microbiol Biotechnol 108:370. doi:10.1007/s00253-024-13210-6 38861018 PMC11166776

[8] Biessy A, Filion M. 2024. Complete genome sequence of Bacillus pumilus LBUM494, a plant-beneficial strain isolated from the rhizosphere of a strawberry plant. Microbiol Resour Announc 13:e0082524. doi:10.1128/mra.00825-24 39248540 PMC11465737

[9] Andrews S. 2017. FastQC: a quality control tool for high throughput sequence data. Babraham Bioinformatics. https://www.bioinformatics.babraham.ac.uk/projects/fastqc/

[10] Tatusova T, DiCuccio M, Badretdin A, Chetvernin V, Nawrocki EP, Zaslavsky L, Lomsadze A, Pruitt KD, Borodovsky M, Ostell J. 2016. NCBI prokaryotic genome annotation pipeline. Nucleic Acids Res 44:6614–6624. doi:10.1093/nar/gkw569 27342282 PMC5001611

[11] Meier-Kolthoff JP, Göker M. 2019. TYGS is an automated high-throughput platform for state-of-the-art genome-based taxonomy. Nat Commun 10:2182. doi:10.1038/s41467-019-10210-3 31097708 PMC6522516

[12] Richter M, Rosselló-Móra R, Oliver Glöckner F, Peplies J. 2016. JSpeciesWS: a web server for prokaryotic species circumscription based on pairwise genome comparison. Bioinformatics 32:929–931. doi:10.1093/bioinformatics/btv681 26576653 PMC5939971

[13] Sievers F, Wilm A, Dineen D, Gibson TJ, Karplus K, Li W, Lopez R, McWilliam H, Remmert M, Söding J, Thompson JD, Higgins DG. 2011. Fast, scalable generation of high-quality protein multiple sequence alignments using clustal omega. Mol Syst Biol 7:539. doi:10.1038/msb.2011.75 21988835 PMC3261699

[14] Price MN, Dehal PS, Arkin AP. 2010. FastTree 2--approximately maximum-likelihood trees for large alignments. PLOS ONE 5:e9490. doi:10.1371/journal.pone.0009490 20224823 PMC2835736

[15] Shimodaira H, Hasegawa M. 1999. Multiple comparisons of log-likelihoods with applications to phylogenetic inference. Mol Biol Evol 16:1114–1116. doi:10.1093/oxfordjournals.molbev.a026201

